# The Effects of Elastic Band Exercises with PNF on Shooting Speed and Accuracy in Ball Hockey Players during the COVID-19 Pandemic

**DOI:** 10.3390/ijerph182111391

**Published:** 2021-10-29

**Authors:** Dagmar Pavlů, Aneta Škripková, David Pánek

**Affiliations:** Faculty of Physical Education and Sport, Charles University, 16252 Prague, Czech Republic; anet.skripkova@gmail.com (A.Š.); panek@ftvs.cuni.cz (D.P.)

**Keywords:** COVID-19, ball hockey, shooting accuracy, shooting speed, proprioceptive neuromuscular facilitation, exercises, elastic resistance

## Abstract

The coronavirus pandemic has affected life and left one of the strongest negative effects on sport. The aim of our study was to evaluate how a simple exercise performed with elastic resistance during the COVID-19 pandemic, when athletes cannot train, affects the basic shooting characteristics of ball hockey players. Extra-league ball hockey players (*N* = 30, age 19–37 years) were randomly divided into an experimental group, which performed elastic resistance exercises with Proprioceptive Neuromuscular Facilitation (PNF) elements for eight weeks, and a control group, which did not perform any exercises. Before the start of the experiment and after it was completed, the speed and accuracy of shooting were measured. In experimental group, there was no decrease after 8 weeks in the shooting speed, and in the control group, there was a statistically significant decrease. There was a deterioration in the accuracy of shooting in both groups; however, in the experimental group, the deterioration was not significant. The results show that even three simple exercises with elastic resistance according to the PNF concept performed 10 times per day for eight weeks can maintain the level of basic skills of ball hockey players—the speed and accuracy of shooting—even when no other training is performed.

## 1. Introduction

The coronavirus pandemic has profoundly affected life and everything around us relating to it—worldwide industry, transport, tourism, education, and leisure. The pandemic has also left one of the strongest negative effects on sport at all levels. The world of sport has been affected by several measures, and it is possible to assume that these measures will continue to limit sport for some time. Nearly all sports venues have been closed, it has not been possible to perform standard training, and sports competitions of various levels including the Olympic Games have been canceled, with a significant negative impact [1,2,3].

Several studies have addressed the negative effects of the pandemic on the lives of the sporting population, both in relation to the younger population [4,5] (e.g., Center for Sport and Human Rights 2020, Ron Gilat) and adults [2], and of course on athletes at various different performance levels [6]. In relation to the lack of physical activity, broader health consequences have been highlighted, with negative impacts on a range of body functions [7,8].

The pandemic has also affected team sports, where most teams, despite the Government restrictions, have placed an emphasis on “being active” and are therefore trying to maintain a certain level of training and prevent a reduction in fitness levels [9,10,11]. Not only in team sports but also in other sports as well are methods to cope with the impact of the pandemic being sought, including the issuing of recommendations [12].

Ball hockey, the international federation of which brings together 54 countries across all continents, is a popular team sport in many countries [13]. Ball hockey is also one of the most popular sports in the Czech Republic, and it is the sixth most practiced team sport. The basic skills of players, which are focused on during regular training, include the speed and accuracy of shooting. In the times of the pandemic and the inability to train, methods have been and still are being sought to help keep players fit, especially through training that can be performed outside of sports facilities, individually and within a home environment [14,15].

Shooting is one of the most important activities in hockey. Shooting is influenced to some extent by the speed and strength of the player and the level of maximum strength achieved [16]. In order to achieve optimal shooting, it is not enough to practice this skill, but it is also necessary to develop muscle strength, speed and endurance [17].

The use of elastic resistance in the form of elastic bands is a simple way of exercising at home due to its ease of use, affordability, and usability virtually anywhere [18,19]. Exercising with elastic resistance is simple, easy to perform, dispensable, and can be used for various purposes such as strength training, the training of coordination skills, exercises to improve range of motion, etc. [20].

The objective of our study was to evaluate how simple elastic resistance exercises with Proprioceptive Neuromuscular Facilitation (PNF) elements, performed in a home environment during the COVID-19 pandemic when athletes cannot train, affect the basic shooting characteristics of ball hockey players—i.e., the speed and accuracy of shooting. We assumed that simple exercises with elastic resistance would prevent a decline in the basic shooting characteristics of speed and accuracy.

## 2. Materials and Methods

### 2.1. Subjects

The basic characteristics of the participants are given in Table 1. All male ball hockey players of one of the largest extra-league teams in the Czech Republic (there are a total of 12 extra-league teams in the Czech Republic) who met the set criteria were included in the study.

Players (*N* = 30) aged 25.40 ± 5.14 years voluntarily participated in the study, who were randomly divided into an experimental group, which performed exercises with elastic resistance (*N* = 15), and a control group, which did not perform any exercises (*N* = 15). All of the participants in the period before the onset of measures relating to the COVID-19 pandemic were medically fit to play and performed the same training program throughout the year.

The criteria for non-inclusion in the study were conditions after serious injuries or operations in the upper limbs, as well as infectious diseases and convalescence after illness or injury.

All of the participants were informed of how the experiment would proceed, and they signed an informed consent. The experiment was approved by the Ethics Committee of Charles University FTVS (EC UK FTVS No. 137/2020).

### 2.2. Procedures

Before the start of the experiment (September 2020), the speed and accuracy of shooting were measured in both groups. The participants of the experimental group were trained to perform elastic resistance exercises with PNF elements, which they performed once per day for eight weeks. The control group did not perform any exercise. After eight weeks (November 2020), the shooting speed and accuracy were measured again in both groups.

#### 2.2.1. Shooting Speed

The shooting speed was measured using a sports radar (Supido Multi Sports Personal Speed Radar). All of the participants shot by hand with a short swing from a distance of 10.50 m from the goal line; i.e., from the line joining the top of the face-off circles [21]. Each participant shot three times, and the average was calculated from the measured values.

#### 2.2.2. Shooting Accuracy

A tarpaulin training sheet with four holes in the corners of the goal and one hole in the middle in the lower half of the goal was used to evaluate the accuracy of shooting. Players were filmed with a camera during the experiment. Each participant had two attempts to hit each of the holes; i.e., each participant had a total of 10 shots. To evaluate the accuracy of shooting, a point rating was chosen as used in the study by Martini et al. [22].

#### 2.2.3. Exercises with Elastic Resistance with Proprioceptive Neuromuscular Facilitation Elements

For the exercises, an elastic band (extensibility from 100% to 300% of the length/3.2–4.5 kg) commonly used in sports [20] and movements from the Proprioceptive Neuromuscular Facilitation (PNF) concept [23], which correspond to the movements performed when shooting in street hockey, were chosen. When shooting, one upper limb performs the first flexion diagonal, while the other upper limb performs the second flexion diagonal. The extension and rotation of the upper torso correspond to the complex performance of shooting.

**Figure 1:**
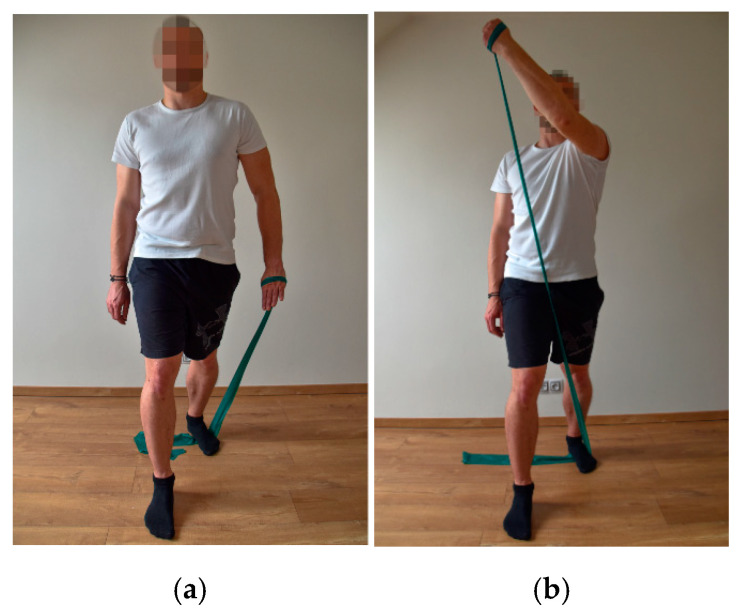
First flexion diagonal—starting position (**a**) and end position (**b**).

**Figure 2:**
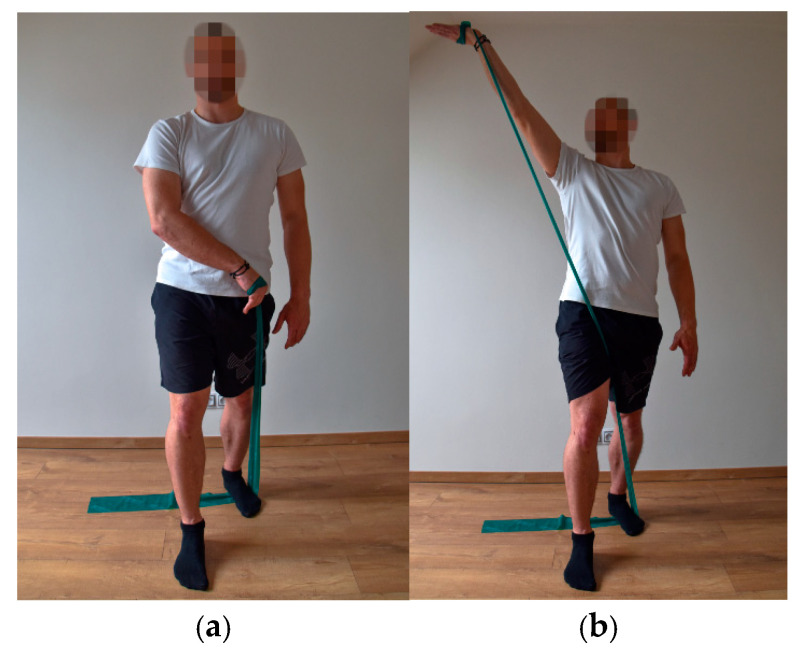
Second flexion diagonal—starting position (**a**) and end position (**b**).

**Figure 3:**
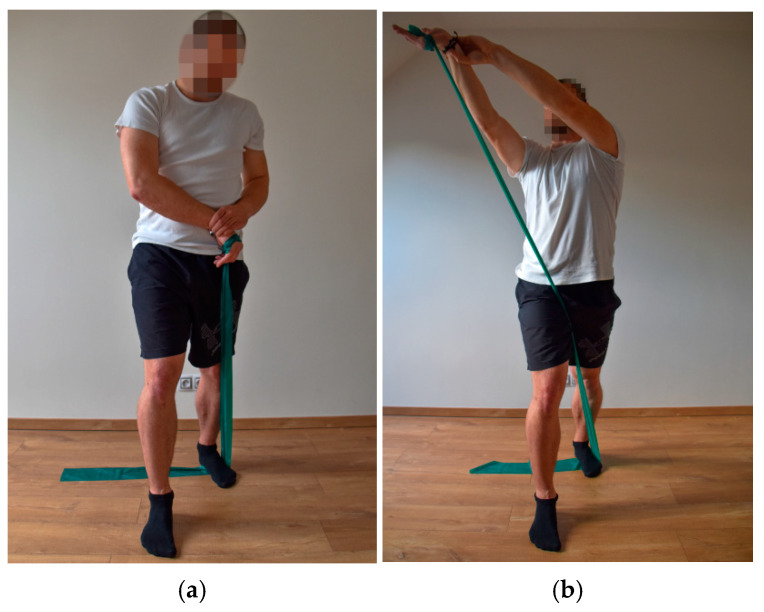
Extension and rotation of the upper torso—starting position (**a**) and end position (**b**).

The participants performed a total of three exercises, always on the right and left side of the body (first flexion diagonal on the upper limb, second flexion diagonal on the upper limb, extension and rotation of the upper torso) [23]. All of the exercises were performed in a standing position with a slow, smooth movement; i.e., from the starting position, there was a concentric contraction against the resistance of the elastic band, and when returning to the starting position, an eccentric contraction began. Each exercise was performed 10 times in two series (Figure 1, Figure 2 and Figure 3). After each series, a break of 60 s was included. After 6 weeks, to eliminate load adaptation, the number of series was increased from 2 to 3, with a break period of 60 s after each series. This number of repetitions was chosen based on the repetition maximum [24]. The maximum number of repetitions was determined as the number of repetitions, when the exercise was performed exactly, without any bends, smoothly, in the full range of motion, and without shaking. Since respondents were players with the same performance level in the off-cover time, they all achieved the same number of repetitions.

All participants from the experimental group performed exercises at the same time of the day—in the afternoon. Prior to the exercise, each athlete was individually instructed to perform the exercise and received an instructing video with the correct execution of all exercises. During the experiment, the physiotherapist regularly checked each participant by phone or in an online meeting. Participants in both the control and experimental groups had the same daily routine. At the time of the experiment—at the time of the COVID-19 pandemic—the respondents were working from home and engaged in office activities. They did not perform any physical activities other than common housework.

### 2.3. Data Processing

The obtained data were analyzed and compared in the statistical program R [25]. In order to determine the distribution of data, the Shapiro–Wilk test of normality and the standardized Breusch–Pagan test of homoscedasticity of residues were used. The paired t-test and two-sample Welch’s t-test were performed to compare the significance of differences, and the determined level of significance was α = 0.05. Cohen’s d (Effect Size) was used to assess clinical significance. A small size of the effect corresponds to values in the range from 0.20 to 0.49. Values from 0.50 to 0.79 indicate a medium size of the effect, and when values equal to or greater than 0.80 are reached, the effect size can be marked as large [26].

Null hypotheses for data processing in the form of H0, “mean values of differences in the speed/accuracy of shooting from input and output measurements are the same for the control and PNF group”, were compared to the two-sided alternative. In this case, the calculated values of the tested statistics were equal to the following series: speed = −3.763 and accuracy = −0.889. In the first case, with respect to a *p*-value equal to 0.0009, the null hypothesis at any reasonable level of reliability α was rejected. In accordance with general practice, α = 0.05 was taken for further tests. The 95% confidence interval for the difference in rate change between the control and PNF groups before and after the intervention was in the interval (−8382; −2462). Thus, for the PNF group, a statistically significantly larger difference, a smaller decrease, or a larger increase in shooting rates between the values at the beginning and end of the specified time period is visible.

In the second case—i.e., the case of shooting accuracy—a *p*-value for the test equal to 0.381—i.e., greater than the selected confidence level—was achieved. As a result, the test does not show the invalidity of the null hypothesis, and there is no statistically significant difference between the groups in the case of shooting accuracy with respect to the chosen test.

## 3. Results

All 30 ball hockey players completed the experiment. The results, evaluated by a paired *t*-test and Cohen’s d (Effect Size), showed significant changes in the evaluated parameters between the experimental and control groups.

### 3.1. Changes in Shooting Speed

**Figure 4:**
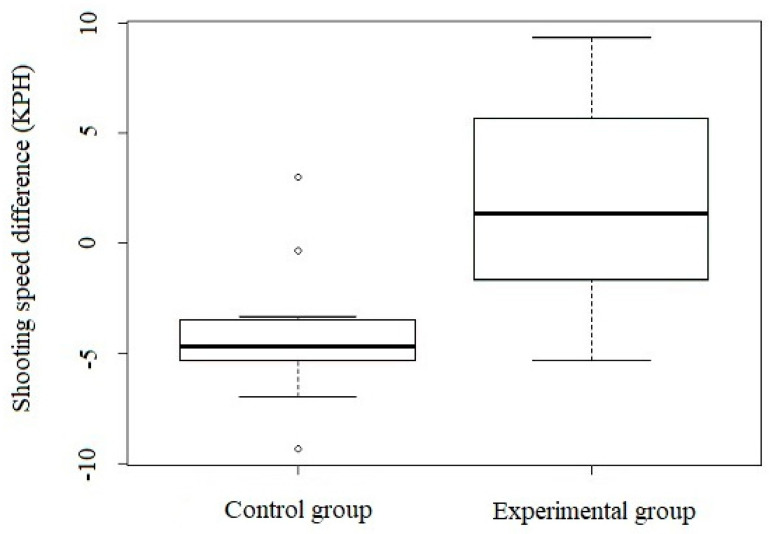
Changes in shooting speed before and after the intervention (control group *n* = 15, experimental group *n* = 15); horizontal line in the box = median; vertical line = data variability outside the upper and lower quartiles. Intermittent points indicate outliers.

Figure 4 shows the changes in shooting speed before and after the intervention. In the experimental group (Table 2), there was no statistically or clinically significant deterioration in the shotting speed after eight weeks compared to the control group (Table 2), in which there was a statistically and clinically significant decrease in this ability; i.e., a significant deterioration of shooting speed.

### 3.2. Changes in Shooting Accuracy

**Figure 5:**
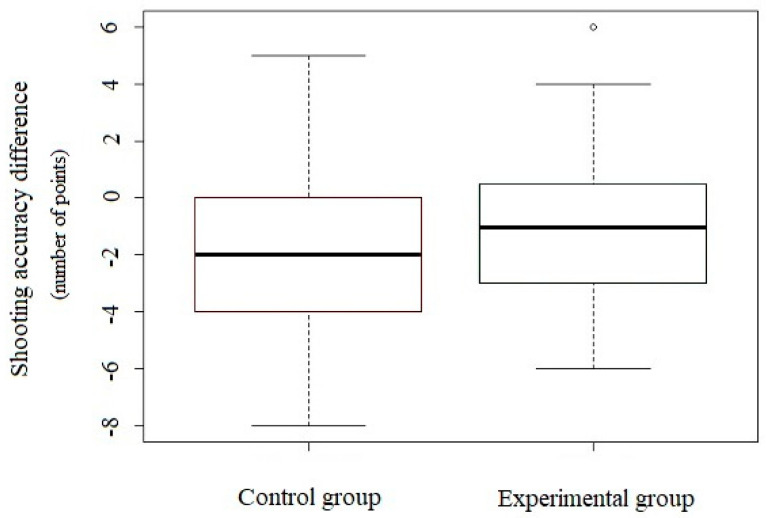
Changes in shooting accuracy before and after the intervention (control group *n* = 15, experimental group *n* = 15); horizontal line in the box = median; vertical line = data variability outside the upper and lower quartiles. Intermittent points indicate outliers.

Figure 5 shows the changes in shooting accuracy before and after the intervention. In both groups (Table 2), the shooting accuracy deteriorated, but in the control group (i.e., the group that did not performed the exercises), the deterioration was significant—the value of the effect size was at the medium level.

## 4. Discussion

The aim of our study was to evaluate how a simple exercise performed in a home environment with elastic resistance during the COVID-19 pandemic, when athletes cannot train, affects the basic shooting characteristics of ball hockey players; i.e., the speed and accuracy of shooting. Our hypothesis that simple exercises with elastic resistance can prevent a decrease in shooting speed and accuracy has been confirmed.

The results showed that players who did not train at all for eight weeks but performed simple elastic resistance exercises with PNF elements did not show a decrease in shooting speed compared to the group that did not exercise and for whom there was a significant decrease in shooting speed. The evaluation of shooting accuracy showed that there was a deterioration in both groups, but in the group that performed the exercises with elastic resistance, the deterioration was not statistically significant.

We consider our study to be unique because no similar study focusing on preventing the loss of speed and accuracy of shooting in hockey or other related sports has been found in the available literature so far.

During our experiment, we searched for ways of helping athletes who cannot train during lockdown. Therefore, we used a simple exercise with elastic resistance that was simple, non-vigorous, and could be used in a home environment. Our idea turned out to be a good one, and the results of performing the exercises with elastic resistance showed that it is a suitable means of maintaining at least a certain level of skill in athletes—in our case, ball hockey players. This is consistent with the results of several other studies that show the positive effects of elastic resistance exercises on functional fitness and strength [16], but probably also because elastic resistance exercises lead to the same results as traditional strength training [19] or, as reported by Areas et al. [27], exercise with elastic resistance affects not only the function of the upper limbs but also other functions such as respiration.

It is well known that systematic strength training leads to structural and functional changes and/or adaptation [27]. Our intervention with elastic resistance for eight weeks may be considered as systematic training, despite the fact that it was a simple exercise. This is partly because there is evidence that the ability of muscles to produce strength has a strong effect on the stability of coordination of certain movements [28]. Therefore, it is possible to assume that the training we applied should help to improve motor coordination. This may also be supported by other authors who have addressed the influence of coordination in relation to training [29,30]. Salehzadeh et al. [31] also demonstrated the relationship between strength training on the one hand and reaction time, neuromuscular coordination, and goal tracking on the other. However, their intervention in a group of 15 athletes lasted 12 weeks and was performed only three times a week, compared to our intervention which lasted eight weeks with daily exercise.

In our study, we intentionally used Proprioceptive Neuromuscular Facilitation (PNF) movements for the elastic resistance exercises, which are characterized by diagonal movements, because these diagonal movements correspond to movements typical of the activities performed and trained by street hockey players. The effects of PNF have been addressed in many studies, a systematic review of which has been made, for example, by Opplert et al. [32]. Mostly positive effects on the development of physical fitness have been reported; i.e., flexibility, strength, balance, endurance, but also sprint performance [33]. Comel et al. [34] add that the PNF method promotes the greater use of dynamic stabilizing shoulder muscles during exercises with the diagonal elevation of the upper limbs. To date, however, not many studies have verified in detail the mechanisms of action of the PNF technique. Nevertheless, our results may be supported by the results of a study conducted by Hindle et al. [35], which confirmed the expected effects of the applied procedure; i.e., an increase in range of motion, strength, and athletic performance. However, based on our practical experience, our results also point to the fact that Proprioceptive Neuromuscular Facilitation has an effect only if it is performed correctly and consistently. Our experiment placed great emphasis on the correct and consistent implementation of the procedures.

After eight weeks of the intervention, the accuracy of shooting deteriorated. To time-limit data collection and at the same time prevent fatigue in our study, players fired 13 shots. A similar number of shots was used in the study by Robbins et al. [36] for ice hockey players. It is possible to discuss whether it would be appropriate to include a higher number of shots to increase the quality of the results or whether there would be signs of fatigue in players when extending data collection. Most studies use a higher number of attempts to evaluate shooting skills in different sports [37,38,39]. However, some studies have also focused on a lower number of shots to evaluate sports performance [21,40,41].

We consider our work to be very beneficial and possibly the first to show that, even in the time of lockdown, when it is not possible for athletes to train, there are simple exercise options to reduce some of the negative effects of the absence of training.

Last but not least, we believe that similar exercises can be used in the general population in order to influence or maintain levels of strength and coordination skills. However, further studies will be needed to confirm this assumption.

We are well aware of the fact that our study also has its limitations. The training only lasted eight weeks, and research with a long-term consistent intervention would be appropriate. The study was only performed on ball hockey players from a single team, which is an advantage on the one hand, but for a generalization for all ball hockey players, other studies with players from different teams seems appropriate. Last but not least, only two skills—speed and accuracy of shooting—were evaluated, whereas muscle strength and range of motion were not included. Our aim was not to evaluate the influence of BMI, age, and duration of sports activities on the monitored parameters, but we are aware of the fact that these covariates could also be reflected in shooting speed and accuracy. Another limitation is the fact that, in our sample, we did not evaluate the mental fatigue that was observed in some athletes during the COVID-19 pandemic [42]. It is necessary to take into account the fact that mental fatigue can affect the shooting abilities of athletes in team sports [43].

## 5. Conclusions

Our results show that three simple exercises with an elastic resistance band using elements of the PNF concept performed 10 times every day for eight weeks leads to the maintenance of the level of basic skills of ball hockey players, which is reflected in the speed and accuracy of shooting, when no other training is performed. A simple exercise with elastic resistance seems to be a suitable way of eliminating the effect of the absence of training and the decrease in the performance of a given sports activity.

## Figures and Tables

**Table 1:** Basic characteristics of participants (*n* = 30).

Variable	Experimental Group		Experimental Group	
	Mean ± SD	Median	Mean ± SD	Median
Age (years)	25.40 ± 5.14	24	24.00 ± 3.76	24
Body mass (kg)	84.90 ± 7.14	85	81.80 ± 10.60	81
Height (cm)	183.00 ± 6.34	185	182.33 ± 5.87	181
BMI (kg/m^2^)	25.35± 1.66	24.68	24.59 ± 2.86	25

**Table 2:** Speed and accuracy of shooting: average values at the beginning and after 8 weeks of intervention—experimental group and control group.

Variable	Experimental Group (*N* = 15)				Control Group (*N* = 15)			
	PRE	POST			PRE	POST		
	Mean ± SD	Mean ± SD	*p*-Value	ES	x ± SD	x ± SD	*p*-Value	ES
Speed of shooting (km/h)	117.78 ± 10.55	119.40 ± 9.03	0.178	0.379	111.60 ± 12.38	107.80 ± 12.29	0.001 *	1.157 “
Accuracy of shooting (points)	4.47 ± 2.16	3.80 ± 2.45	0.462	0.202	4.67 ± 2.33	2.93 ± 2.54	0.051	0.570

PRE = before the experiment; POST = after 8 weeks; ES = effect size; * statistically significant decrease—level of significance α = 0.05; “clinically significant decrease.

## Data Availability

The datasets generated for this study are available on request to the corresponding author.
